# A Case of Carbamazepine-Induced Aggravation of Self-Limited Epilepsy with Centrotemporal Spikes Epilepsy and Valproate-Induced Hyperammonemic Encephalopathy in a Child with Heterozygous Gene Variant of Carbomoyl Phosphatase Synthetase Deficiency

**DOI:** 10.1155/2021/2362679

**Published:** 2021-12-31

**Authors:** Imalke Kankananarachchi, Eresha Jasinge, Gemunu Hewawitharana

**Affiliations:** ^1^Department of Paediatrics, Faculty of Medicine, University of Ruhuna, Matara, Sri Lanka; ^2^Lady Ridgeway Hospital, Colombo, Sri Lanka; ^3^Teaching Hospital Karapitiya, University of Ruhana, Matara, Sri Lanka

## Abstract

Antiepileptics drugs are the mainstay of the management of epilepsy in children. Sodium valproate (VPA) and carbamazepine (CBZ) are widely used medications in childhood epilepsy. Hyperammonemia has been described as a known side effect of valproate therapy. It is known that VPA-associated HA is common among patients who hold genetic mutations of the carbomoyl phosphatase synthase 1 gene (CPS1). Aggravation of self-limited epilepsy with centrotemporal spikes (SLECTS) is a rare side effect of CBZ. Here, we present a child who had CBZ-induced aggravation of rolandic epilepsy and VPA-induced HA encephalopathy in the background of an unrecognised heterozygous gene variant of CPS1. An 8-year-old boy with SLECTS presented with a history of abnormal behaviours and drowsiness. He was apparently well until six years when he developed seizures in favour of rolandic epilepsy. His electroencephalogram (EEG) showed bilateral predominantly on the right-sided central-temporal spikes and waves. The diagnosis of SLECTS was made, and he was commenced on CBZ. Though he showed some improvement at the beginning, his seizure frequency increased when the dose of CBZ was increased. His repeat EEG showed electrical status in slow-wave sleep, and CBZ was stopped. Subsequently, he was started on VPA, and with that, he developed features of encephalopathy. He had elevated serum ammonia with normal liver functions. VPA was stopped with the suspicion of VPA-induced hyperammonemia. Tandem mass spectrometry did not show significant abnormality in the amino acid profile. Specific genetic analysis revealed a c.2756 C > T.p (Ser919Leu) heterozygote genetic mutation of the CSP 1 gene. This is a classic example where side effects of treatment determine the choice of antiepileptics drugs (AEDs) in childhood epilepsy. It is essential to keep in mind that SLECTS can be aggravated with certain AEDs, and VPA-induced HA in the absence of live failure could be due to underlying inherited metabolic disorders.

## 1. Introduction

AEDs are the mainstay of the management of epilepsy in children. However, in 15% of the time, AEDs are withheld due to their adverse effects [1]. Sodium valproate has a wide range of indications in childhood epilepsy. Moreover, it is being used as a mood stabiliser and antimigraine treatment [1]. Hyperammonemia (HA) has been described as a known side effect of valproate therapy even in the absence of underlying liver disease [2, 3]. It has been described that valproate-associated HA is more common among patients who hold genetic mutations of the CPS1 gene [4, 5]. Carbamazepine (CBZ) is a widely used drug in both partial and generalised epilepsy. Neurological side effects are seen in 50% of patients receiving CBZ [6]. Aggravation of self-limited epilepsy with centrotemporal spikes (SLECTS) is a rare side effect of CBZ [7]. Here, we present a child who had carbamazepine-induced aggravation of rolandic epilepsy and valproate-induced hyperammonemic encephalopathy in the background of unrecognised heterozygous gene variant of carbomoyl phosphatase synthetase deficiency.

## 2. Case Presentation

An 8-year-old boy with self-limited epilepsy with centrotemporal spikes (SLECTS) was admitted to the paediatric ward for further evaluation of abnormal behaviours and drowsiness. He was the first child born to nonconsanguineous healthy parents following an uneventful antenatal period. He was born at term via normal vaginal delivery with a birthweight of 3.1 kg. He did not require any resuscitation at birth. The child was well until six years of age when he developed seizures. The semiology of the seizures was predominantly focal motor hemifacial tonic-clonic movements with oropharyngo symptoms and associated with speech arrest and hypersalivation. Occasionally, he had bilateral tonic-clonic seizures as well.

**Figure 1:**
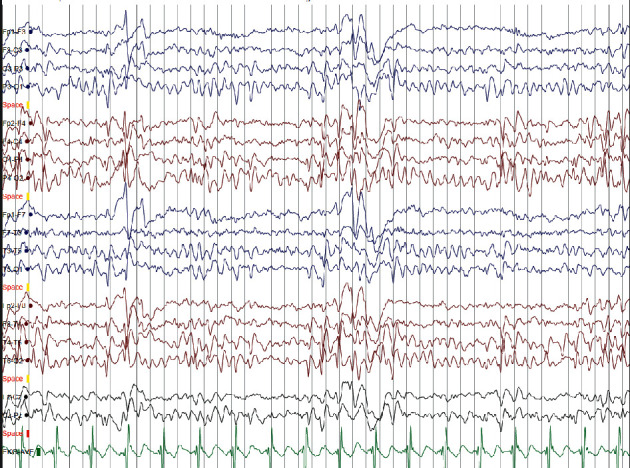
EEG showing predominant centrotemporal spikes and waves on the right side (voltage 70 mV/cm).

His electroencephalogram (EEG) showed high voltage, diphasic spikes with slow activity predominantly on the right central-temporal region, creating a horizontal dipole with maximal electronegativity in the right centrotemporal region (Figure 1). The diagnosis of SLECTS was made based on the clinical and EEG features.

**Figure 2:**
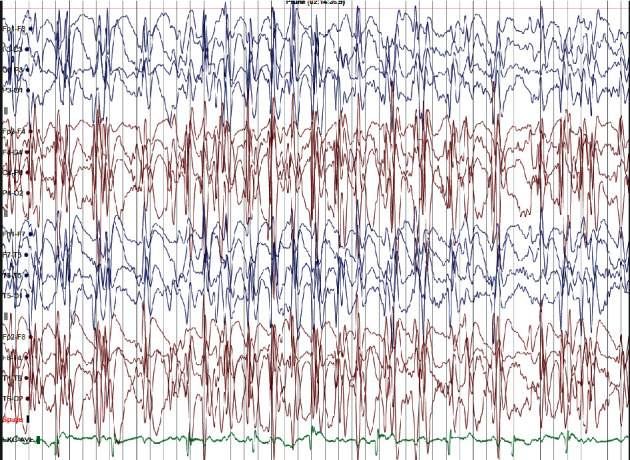
Abnormal EEG showing electrical status following increased carbamazepine dose (voltage 70 mV/cm).

His brain MRI scan and basic biochemical tests were normal. Initially, the family was reassured about the prognosis of the condition, and the decision was taken to manage conservatively without starting AEDs. However, he continued to have at least one seizure fortnightly, and due to extreme parental concerns, he was commenced on CBZ 15 mg/kg/day. He had good seizure control for about one year after the commencement of CBZ. Subsequently, the CBZ dose was increased to 22 mg/kg/day since his seizure frequency increased. However, he had a poor response to CBZ where there was an increased seizure frequency to 1-2 seizures per week and behavioural changes such as violent and aggressive behaviour towards parents and peers. The repeat sleep EEG showed almost continuous spikes and wave activity suggestive of electrical status in slow-wave sleep, and the diagnosis of nonconvulsive status was established (Figure 2). However, there was no history of developmental regression.

**Figure 3:**
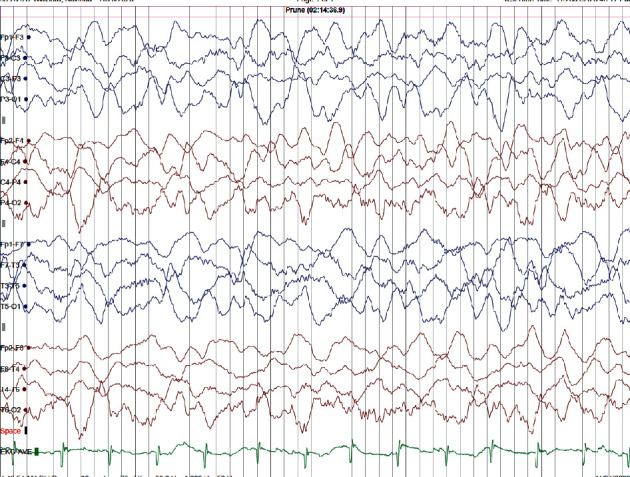
Abnormal EEG showing features of encephalopathy following sodium valproate (voltage 70 mV/cm).

Subsequently, he was started on sodium valproate (VPA) 10 mg/kg/day dose while tailing off CBZ. After a few weeks of staring VPA, his seizures were stopped, but the child became drowsy and encephalopathic. There was a significant change in his behaviour where he became very aggressive and violent. Moreover, he was disoriented for time, place, and person. He had recurrent episodes of vomiting and ataxia but did not go into the level of coma. A repeat EEG was carried out during the drowsiness period and showed generalised high amplitude delta activity without frank evidence of epilepsy was in favour of evolving encephalopathy (Figure 3). His neurological examination was normal except for having a low Glasgow Coma Scale of 13. No abnormality was noted in the basic biochemical investigations, including liver enzymes and blood urea levels. A high degree of clinical suspicion of VPA-induced HA was there at this point, and he underwent second-line investigations. His serum ammonia level was 82.0. Plasma amino acid analysis was carried out using high-performance chromatography postcolumn derivatisation with ninhydrin. The analysis revealed mildly elevated arginine 167 *µ*mol/L (6–140) and serine198 *µ*mol/L (58–187). VPA-induced HA was diagnosed, and he was started on levetiracetam while tailing off VPA. Serum VPA level was not measured due to the unavailability of the test. He showed a marked improvement after stopping VPA. Specific genetic analysis revealed a c.2756 C > T.p (Ser919Leu) heterozygote genetic mutation of the CSP 1 gene. He was regularly followed up at the paediatric neurology clinic for more than a year, and he remained seizure-free while on levetiracetam.

## 3. Discussion

Though SLECTS carries an excellent prognosis, some clinicians start these children on AEDs depending on the clinical picture. However, a few antiepileptics such as CBZ and phenobarbitone result in electroclinical aggravation of the condition [7]. The increasing frequency of seizures, development of atypical absence seizures, speech difficulties, deterioration of school performances, and continuous spikes and waves during slow sleep are recognized as adverse effects of CBZ in SLECTS [7]. Furthermore, CBZ is known to exacerbate some cases of benign occipital epilepsy in childhood [8]. In this case, the child got worsen following the commencement of CBZ, but there were no other side effects noted. As the second-line therapy, the child was started on VPA while tailing off CBZ.

Carbomoyl phosphate synthetase-1 (CPS1) enzyme is responsible for the urea cycle's first and rate-limiting step. CPS1 deficiency (CPS1D) is a rare form of urea cycle defect (UCD), which has autosomal recessive inheritance [4]. The CPS1 gene, located in 2q35 consists of 4500 nucleotide coding sequences scattered through 38 exons, separated by 37 intervening introns [9]. Habre et al. in 2011 described 192 new gene changes of CPS1, amounting to 222 mutations altogether [10]. The CPS1 variant c.2756 C > T.p (Ser919Leu) has been identified as a cause for CPS1D in a few studies [10, 11]. This child was found to have the heterozygous CPS1 variant c.2756 C > T.p (Ser919Leu), and VPA-induced HA is likely to be secondary to the identified genetic mutation.

The precise mechanism of VPA-induced encephalopathy is not well known; however, the accumulation of toxic metabolites of VPA and HA is a possible explanation [2]. Moreover, VA-induced coma is more common when it is given in combination with other AEDs [2]. In this case, VPA was given while tailing off CBZ; therefore, the child was on two antiepileptics when he developed hyperammonemia (HA), which would have contributed to developing HA up to a certain degree. CPS1D has been identified as an underlying reason for VPA-induced encephalopathy in many case reports and series [5, 12]. Piotr et al., in 2013, did ammonia levels of 142 adults who were on VPA of more than 1-year duration and showed that 7.7% of them had elevated ammonia levels. Moreover, the study revealed that those who carry heterozygous T1405 N gene mutation of CPS1 have a higher incidence of HA [13]. In this child, the identified gene mutation was c.2756 C > T.p (Ser919Leu). This mutation was previously described by Harber et al. as one of 222 gene mutations of the CPS1 gene [10]. However, VPA-induced HA in the background of this gene mutation was not previously reported. Though urea cycle defects (UCD) can present with epilepsy, it is not known whether heterozygous individuals get epilepsy [14]. Furthermore, we could not find any association between SLECTS and UCD. Therefore, we do not believe whether there was a link between this identified gene mutation and the epilepsy of this child.

## 4. Conclusion

This is a classic example where the side effects of treatment determine the choice of AEDs in childhood epilepsy. It is essential to keep in mind that SLECTS can be aggravated with certain AEDs; therefore, the risk versus benefit of AEDs in SLECTS should be measured before starting on AEDs. Moreover, VPA-induced HA in the absence of live failure could be due to underlying diseases such as UCDs.

## Data Availability

The data used to support the findings are included within the article.

## Abbreviations

VPA: Sodium valproate
CBZ: Carbamazepine
CPS: Carbomoyl phosphatase synthase
CPS1D: Carbomoyl phosphatase synthase-1 deficiency
SLECTS: Self-limited epilepsy with centrotemporal spikes
EEG: Electroencephalogram
AEDs: Antiepileptic drugs
UCD: Urea cycle defects.
